# Supplementary material for: Macrophage Targeting pH Responsive Polymersomes for Glucocorticoid Therapy
Source: Pharmaceutics. 2019 Nov 15;11(11):614. doi: 10.3390/pharmaceutics11110614 (PMC6920840; doi:10.3390/pharmaceutics11110614)
Supplement: Supplementary file 1 [file pharmaceutics-11-00614-s001.pdf]

pharmaceutics

Supplementary Materials: Macrophage Targeting pH Responsive Polymersomes for Glucocorticoid Therapy

Virgínia M. Gouveia, Loris Rizzello, Claudia Nunes, Alessandro Poma, Lorena Ruiz-Perez, António Oliveira, Salette Reis and Giuseppe Battaglia

Polymers synthesis and characterization ….………………………………………………………...2

Polymersomes characterization study …………………..……………………………..……… 4

Drug release study …………………………………………………………………….………… 5

Cell viability study ……………………………………………………………………..………… 6

Gene expression study ………………………………………………………………...…… 7

Polymer Synthesis and Characterization

PMPC-*b*-PDPA was prepared by loading a round bottom flask (equipped with a magnetic stir bar) with 2-methacryloyloxyethyl phosphorylcholine (MPC, 25 eq.), 2-(4-morpholino)ethyl 2-bromoisobutyrate (ME-Br) initiator (1 eq.) and ethanol (final [MPC] = 2.8M), and this solution was deoxygenated by purging N_2 for at least 1 h under stirring at room temperature. Then, 2,2′-bipyridine (bpy) ligand (2 eq.) and Cu(I)Br (1 eq.) were added as solids whilst maintaining the flask under a mild positive N_2 pressure. The reaction was carried out under a N_2 atmosphere at 30 °C. After 90 min (MPC conversion > 99% from ^{1}H-NMR), an ethanolic solution of 2-(diisopropylamino) ethyl methacrylate (DPA, 85 eq., [DPA] = 3.8 M), previously deoxygenated by purging N_2, was injected into the flask. After 48 h, the reaction solution was opened to air, diluted with ethanol and left stirring for 1 h. The solution was then passed through a silica column to remove the copper catalyst. After this step, the filtrate was concentrated by rotary evaporation and dialysed using a 3.5 kDa MWCO dialysis membrane (Spectrum Labs, Netherland) against chloroform/methanol 2:1 (*v*/*v*) (2–3 × 500 mL), methanol (2 – 3 × 500 mL), and double-distilled water (4 – 6 × 2 L). After dialysis the copolymer was isolated by freeze-drying.

^{1}H-NMR [$CDCl_3$/CD_3OD 3:1 (*v*/*v*), 600 MHz, H given in number per monomer unit, all broad signals]: $PMPC_{25}$-$PDPA_{68}$, δ = 4.24 (2H, PMPC); 4.14 (2H, PMPC) 3.98 (2H, PDPA), 3.84 (2H, PMPC), 3.69 (2H, PMPC), 3.24 (9H, PMPC) 3.00 (2H, PDPA), 2.64 (2H, PDPA), 1.87–1.78 (2H, PMPC and 2H, PDPA), 1.01 (12H, PDPA), 0.89 (3H, PMPC and 3H, PDPA). GPC (H_2O + 0.25% TFA as eluent): $PMPC_{25}$-$PDPA_{68}$, M_n = 21.0 kDa, M_w/M_n = 1.39.

Cy5-labelled PMPC-*b*-PDPA was prepared as above but using bis[2 -(2-bromoisobutyryloxy)ethyl] disulfide as initiator [1]. After purification and isolation, an aliquote of the obtained polymer was reacted with Cyanine5 maleimide (1.1 eq.) and PPh_3 (2 eq.) in degassed chloroform/methanol [2:1 (*v*/*v*)]. The final polymer concentration was 1.6 mM, and the reaction was kept stirring under N_2 and in the dark at room temperature for 48 h. After this time, the reaction solution was opened to the air, filtered onto a silica column and dialysed using a 3.5 kDa MWCO dialysis membrane (Spectrum Labs, Netherland) against chloroform/methanol 2:1 (*v*/*v*) (2 – 3 × 500 mL), methanol (4 – 6 × 500 mL), and double-distilled water (4 – 6 × 2 L). After dialysis the copolymer was isolated by freeze-drying.

GPC (H_2O + 0.25% TFA as eluent): Cy5-$PMPC_{25}$-$PDPA_{70}$, M_n = 23.0 kDa, M_w/M_n = 1.35.

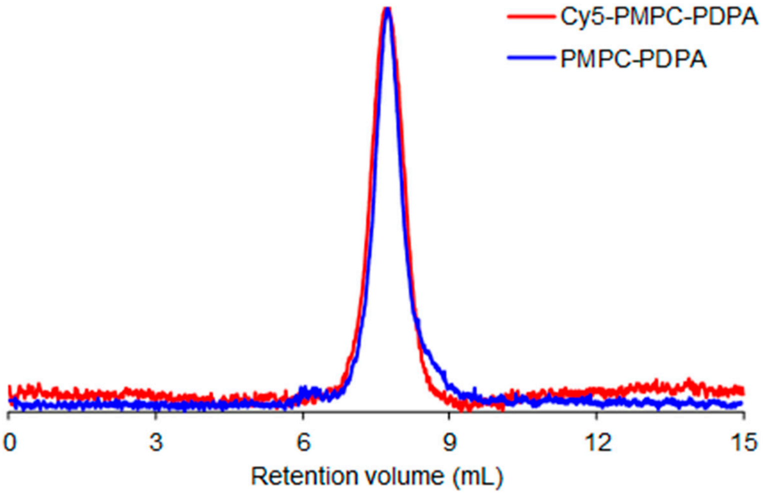

(**a**) (**b**)

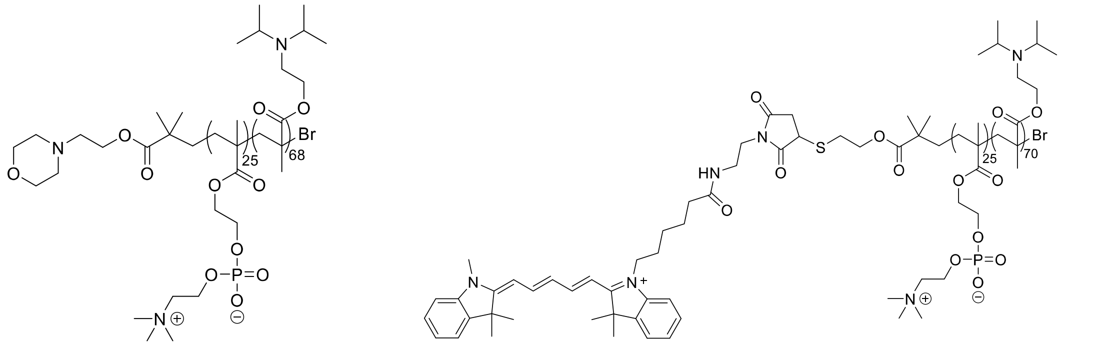

Figure S1: Chemical structure of (**a**) $PMPC_{25}$-$PDPA_{68}$ and (**b**) Cy5-$PMPC_{25}$-$PDPA_{70}$.

Polymersomes Characterization

Regarding the characterization study, HPLC analyses resulted in the drug encapsulation and loading efficiencies within PMPC-PDPA polymersomes. The drug encapsulation efficiency (EE) was calculated as the ratio between the final and initial mass of loaded prednisolone disodium 21-phosphate (PDP). The drug loading efficiency (LE) was determined according to a previously reported method [2] represented as the number of PDP molecules loaded within the total lumen volume of PMPC-PDPA polymersomes (which is related with the size of the vesicle and the actual amount of loaded drug).

(**a**)

	D_h (nm)	PDI
**Psome**	117 ± 5	0.098 ± 0.01
**Psome:PDP**	178 ± 4	0.166 ± 0.10

(**b**)

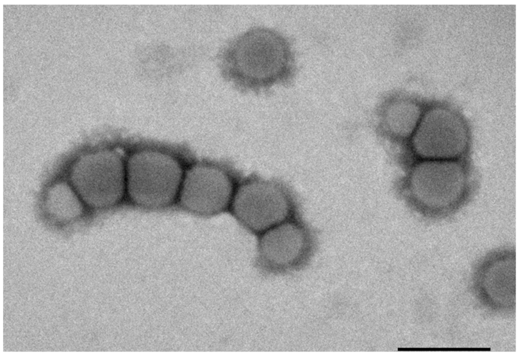

(**c**)

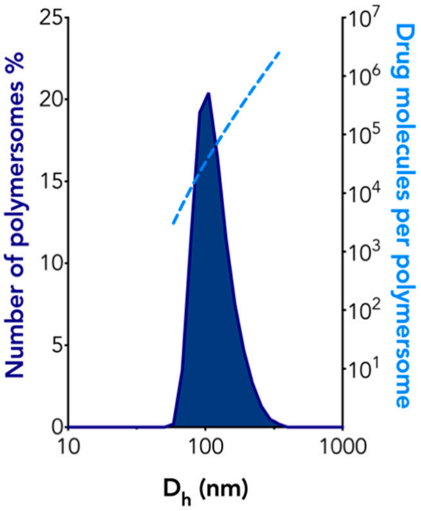

(d)

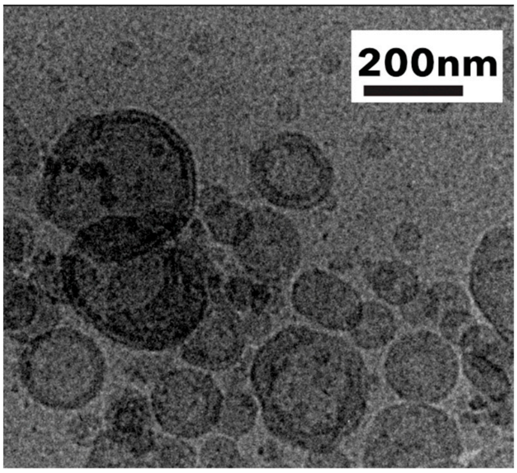

(e)

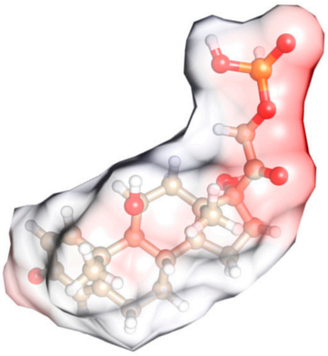

Figure S2: (**a**) DLS data on the hydrodynamic diameter (D_h) and polydispersity index (PDI) values of all formulations of unloaded and PDP loaded PMPC-PDPA polymersomes (n = 3). Analysis on the PDI values below 0.2 indicates a formulation of polymersomes with monodisperse and homogeneous size distribution [3]. (**b**) TEM representative image of Cy5-PMPC-PDPA polymersomes produced via film rehydration method (200 nm scale bar). (**c**) DLS data on the number of PMPC-PDPA polymersomes as a function of the D_h. Analysis on the drug loading capacity represented as the number of PDP molecules per polymersome as a function of their size. (**d**) Cryo-TEM representative image of PMPC-PDPA polymersomes produced via pH-switch method (200 nm scale bar). (**e**) Chemical structure and electrostatic surfaces of prednisolone disodium 21-phosphate (PDP) and respective representation of the electrostatic surfaces.

Drug Release study

To examine the kinetics and mechanism of PDP release from the PMPC-PDPA polymersomes, the data obtained from the in vitro drug release studies of each pH profile was analyzed using various models, including the zero and first order, Higuchi, Hixson-Crowell and Korsmeyer-Peppas models [4,5].

Table S1. Mathematical models for drug-release kinetics.

Release Model	Equation [1]	Information
Zero–Order	$Q = Q_0 + K_0 t$	refers to the process of constant drug release from a drug delivery device
First–Order	$\text{Log } C = \text{Log } C_0 - k_1 t / 2.303$	represents a system where the release rate of the drug depends on the concentration of the drug in the system
Hixson–Crowell	$Q_0 1/3 - Q_t 1/3 = K_{HC}\, t$	describes the release from systems where there is a change in surface area and diameter of particles
Higuchi	$Qt = k_H\, (t)^{0.5}$	assumes that the drug's release is caused primarily by a diffusion mechanism
Korsmeyer–Peppas	$F = M_t/M\ N = Kt^n$	provides insight into the type of drug release mechanism taking place from swellable devices

[1] Q is the amount of drug released or dissolved; Q_0 is initial amount of drug in solution; C_0 is the initial concentration of drug; t is the time in hours; F is the fraction of drug release at time t; M_t/M is the fraction of drug released at time t; K are the rate constants for each models.

Table S2. Correlation coefficient (r^2) from various drug release mathematical models for each pH profile.

	Zero–Order	First–Order	Hixson–Crowell	Higuchi	Korsmeyer–Peppas
pH 5.0	0.935	0.635	0.643	0.995	0.172
pH 6.5	0.984	0.657	0.757	0.959	0.503
pH 7.4	0.636	0.419	0.410	0.758	0.348

Cell Viability Study

(a) **(b)**

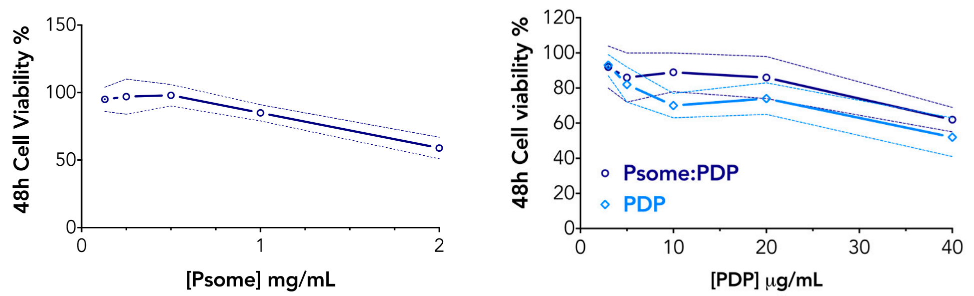

Figure S3: Cell viability assay after 48 h incubation with increasing concentrations of (**a**) unloaded PMPC-PDPA polymersomes, (**b**) either free PDP or PDP-loaded polymersomes (Psome:PDP).

Gene Expression Study

For the RT-qPCR experiments, the ribosomal protein L13A (RPL13A) was used as reference gene, because it was stably expressed in THP-1, both in stimulated and unstimulated cells (data not shown).

Table S3. Forward (Fw) and reverse (Rv) gene sequences of designed primers (PRIMER-BLAS; Sigma-Aldrich) used for gene expression studies.

Gene		Primers	Classification
RPL13A	Fw	CTTCCTTTCCAGTTTGCTGC	ribosomal protein
	Rv	TCTCGCAGTCCACTTCCTTT	
TNFα	Fw	GGAGAAGGGTGACCGACTCA	tumor necrosis factor
	Rv	CTGCCCAGACTCGGCAA	
IL8	Fw	TCCAAACCTTTCCACCCCAAA	chemokine
	Rv	ACCCTCTGCACCCAGTTTTC	
IL6	Fw	TGCAATAACCACCCCTGACC	interleukin
	Rv	AGCTGCGCAGAATGAGATGA	
IL1β	Fw	CCAAAGAAGAAGATGGAAAAGGC	interleukin
	Rv	GGGAACTGGGCAGACTCAAA	

RT-qPCR data was analysed using the comparative cycle threshold (Ct) method, also known as the ΔΔCt method. The Ct value of each target gene (TNFα, IL1β, IL6 and IL8) was normalized to the reference gene (RPL13A), obtaining the ΔCt value (Equation 1) of treatment and control (i.e., non-treated). Then, the change in Ct is compared against the control to obtain the ΔΔCt value (Equation 2) using the following equations:

$$\Delta Ct = Ct\ (\text{target gene}) - Ct\ (\text{RPL13A}) \quad (1)$$

$$\Delta\Delta Ct = \Delta Ct\ (\text{treated}) - \Delta Ct\ (\text{non-treated}) \quad (2)$$

Then, the – ΔΔCt values corresponds to the folds in gene expression change of the treated compared to the non-treated group.

References

1. Gaitzsch, J.; Delahaye, M.; Poma, A.; Du Prez, F.; Battaglia, G. Comparison of metal free polymer-dye conjugation strategies in protic solvents. *Polym. Chem.* **2016**, *7*, 3046–3055, doi:10.1039/c6py00518g.
2. Wang, L.; Chierico, L.; Little, D.; Patikarnmonthon, N.; Yang, Z.; Azzouz, M.; Madsen, J.; Armes, S.P.; Battaglia, G. Encapsulation of biomacromolecules within polymersomes by electroporation. *Angew Chem. Int. Ed. Engl.* **2012**, *51*, 11122–11125, doi:10.1002/anie.201204169.
3. Blanco, E.; Shen, H.; Ferrari, M. Principles of nanoparticle design for overcoming biological barriers to drug delivery. *Nat. Biotechnol.* **2015**, *33*, 941–951, doi:10.1038/nbt.3330.
4. Mircioiu, C.; Voicu, V.; Anuta, V.; Tudose, A.; Celia, C.; Paolino, D.; Fresta, M.; Sandulovici, R.; Mircioiu, I. Mathematical Modeling of Release Kinetics from Supramolecular Drug Delivery Systems. *Pharmaceutics* **2019**, *11*, doi:10.3390/pharmaceutics11030140.
5. Mhlanga, N.; Ray, S.S. Kinetic models for the release of the anticancer drug doxorubicin from biodegradable polylactide/metal oxide-based hybrids. *Int. J. Biol. Macromol.* **2015**, *72*, 1301–1307, doi:10.1016/j.ijbiomac.2014.10.038.

© 2019 by the authors. Submitted for possible open access publication under the terms and conditions of the Creative Commons Attribution (CC BY) license (http://creativecommons.org/licenses/by/4.0/).
